# Design Issues in Small-Area Studies of Environment and Health

**DOI:** 10.1289/ehp.10817

**Published:** 2008-04-25

**Authors:** Paul Elliott, David A. Savitz

**Affiliations:** 1 Small Area Health Statistics Unit, Department of Epidemiology and Public Health, Imperial College London, London, UK; 2 Department of Community and Preventive Medicine, Mount Sinai School of Medicine, New York, New York, USA

**Keywords:** air pollution, chlorination by-products, exposure assessment, extremely low-frequency electromagnetic fields, small-area studies, spatial epidemiology

## Abstract

**Background:**

Small-area studies are part of the tradition of spatial epidemiology, which is concerned with the analysis of geographic patterns of disease with respect to environmental, demographic, socioeconomic, and other factors. We focus on etiologic research, where the aim is to make inferences about spatially varying environmental factors influencing the risk of disease.

**Methods and results:**

We illustrate the approach through three exemplars: *a*) magnetic fields from overhead electric power lines and the occurrence of childhood leukemia, which illustrates the use of geographic information systems to focus on areas with high exposure prevalence; *b*) drinking-water disinfection by-products and reproductive outcomes, taking advantage of large between- to within-area variability in exposures from the water supply; and *c*) chronic exposure to air pollutants and cardiorespiratory health, where issues of socioeconomic confounding are particularly important.

**Discussion:**

The small-area epidemiologic approach assigns exposure estimates to individuals based on location of residence or other geographic variables such as workplace or school. In this way, large populations can be studied, increasing the ability to investigate rare exposures or rare diseases. The approach is most effective when there is well-defined exposure variation across geographic units, limited within-area variation, and good control for potential confounding across areas.

**Conclusions:**

In conjunction with traditional individual-based approaches, small-area studies offer a valuable addition to the armamentarium of the environmental epidemiologist. Modeling of exposure patterns coupled with collection of individual-level data on subsamples of the population should lead to improved risk estimates (i.e., less potential for bias) and help strengthen etiologic inference.

Small-area studies are part of the tradition of spatial epidemiology, which is concerned with the analysis of geographic patterns of disease with respect to environmental, demographic, socioeconomic, and other factors (Elliott and Wartenberg 2004). In this article, we focus on the use of small-area studies in etiologic research, where the aim is to make inferences about spatially varying environmental factors (“exposures”) influencing the risk of disease.

Many environmental exposures, such as those from air or drinking water, are, by their very nature, determined in large part by location. Individual exposure to such pollutants is determined by geographic factors such as where we live, where we work, where we go to school, and the like, as well as how we move through, and interact with, the exposure “surface,” for example, time spent outdoors or amount of water ingested. This, in turn, will reflect individual demographic and lifestyle factors such as age, sex, social class, income, job, and mode of travel, as well as other less readily measured factors that influence the way we lead our daily lives.

For etiologic research, ideally the analysis will be based on individual-level data. Although precise measures of pollutants released into the environment are often available—for example, continuously monitored effluent concentrations in a stack, air pollutant concentrations measured at a fixed-site monitor, levels of disinfection by-products at a sampling point in the distribution of the water supply—this is not the case for measures of individual exposure. Even where feasible, individual-based sampling—for example, passive sampling of nitrous oxides as a marker of ambient pollution from road-traffic pollution (van Roosbroeck et al. 2006)—can be done only for relatively small numbers of people and over short time periods (days or weeks). These often provide estimates of external rather than internal exposure (unless based on biologic sampling, e.g., from blood, urine), whereas from a biologic perspective, we are usually interested in internal dose, perhaps accumulated over long periods (months or years).

Obtaining individual-based measures of exposure is clearly infeasible where many thousands, hundreds of thousands, or even millions of people potentially constitute the “at-risk” population. Instead, we have to rely on modeling of exposures, ranging from simple measures such as distance from a point source (Elliott et al. 1996) or distance to nearest road (Hoek et al. 2002; Wilkinson et al. 1999), to more complex estimation, for example, dispersion modeling around a point source (Bellander et al. 2001; Hodgson et al. 2007). Such proxy measures based on modeling may or may not adequately capture the exposure of the individual to some pollutant or pollutants of concern, leading to possible exposure misclassification.

Exposure misclassification is well known in classical epidemiology and, if nondifferential with respect to disease status, will generally lead to “regression dilution bias” (MacMahon et al. 1990), that is, bias of effect size estimates toward the null; however, this will not always be the case in ecologic regression, that is, where the group rather than the individual is the unit of analysis (Brenner et al. 1992; Webster 2002). Given that the effect of environmental exposures in a well-regulated society is expected to be small—at least in terms of excess relative risk—nondifferential exposure misclassification leading to bias toward the null could lead to false-negative findings and false reassurance about the health effects associated with a particular exposure or exposures. In public health terms, this could be important because even small excess relative risks, if applied to large numbers of people, could result in large numbers of excess cases of disease (Rose 1981). From a local community perspective also, even small numbers of excess cases of rare diseases such as childhood leukemia, or a small increase in the prevalence of adverse reproductive outcomes such as birth defects, could have devastating consequences for the individuals and populations affected. It is therefore important that our exposure measures be as precise and accurate as possible. Differential errors clearly could also lead to bias, often in the opposite direction (i.e., away from the null); for example, the differential reporting of symptoms near a source of environmental contamination may lead to apparent associations with the source, possibly reflecting public perception and concern rather than direct effects of pollution on health per se (Hunter et al. 2004).

We now discuss some of the design issues in small-area epidemiologic research, with particular emphasis on improving exposure classification and reducing bias. We then illustrate some of the strengths and also limitations of small-area analyses, through three exemplars, chosen to illustrate different aspects of the approach: *a*) magnetic fields from overhead electric power lines and the occurrence of childhood leukemia, which illustrates the use of geographic information systems (GIS) to focus on areas with high exposure prevalence; *b*) drinking-water disinfection by-products and reproductive outcomes, taking advantage of large between- to within-area variability in exposures from the water supply; and *c*) chronic exposure to air pollutants and cardiorespiratory health, where issues of socioeconomic confounding are particularly important.

## Design Issues

To be informative, small-area epidemiologic studies should include a population with a wide range of exposure to the environmental contaminants of interest. Spatial location should be an accurate exposure indicator, a sufficiently large population should be included to generate precise estimates of rare health events such as birth defects or childhood cancers that are often of concern, and information on the associated characteristics of the exposed population, that is, confounders, needs to be available. Each of these desirable attributes helps to determine whether the application of spatial methods will be a useful strategy in a particular setting.

The trade-off between the geographic scope of the study and the ability to isolate the environmental agent of interest needs to be considered. Although environmental exposures often differ across geographic areas, there is also a tendency for other disease determinants to differ across areas, as well, because attributes such as socioeconomic status, ethnic composition of the population, and accompanying lifestyle factors (diet, tobacco use) tend to cluster together. To the extent that these variables might be geographically coincident with environmental exposure data, such socioeconomic and lifestyle variables may be powerful spatial confounders and lead to bias if not dealt with adequately in the analysis.

Geographic studies have traditionally been thought of as pure ecologic studies and, as such, have been criticized because inferences at the group level may not hold at the individual level, the “ecological fallacy” (Morgenstern 1995; Piantadosi et al. 1988). In particular, “pure specification bias” (Greenland 1992) arises when there is non-constant within-area exposure distribution and a nonlinear exposure–response relationship, such that the model relating risk to exposure at individual level may differ to that at group level. In addition, there are issues of within- and between-group confounding and effect modification, among other potential sources of bias (Elliott and Wakefield 2000; Wakefield and Salway 2001).

At the extreme, we could generate marked contrasts in exposure by comparing disease rates in places with and without exposure to a particular environmental contaminant. For example, we could compare disease rates in countries in which dichlorodiphenyl-trichloroethane (DDT) is used with those in which it has been banned, but this would lead largely to a contrast between poor and wealthy nations or between southern and northern regions. No matter how much effort might go into isolating the effect of DDT exposure from other influences on disease, such studies are doomed by the array of other concomitant influences on disease that cannot be effectively controlled. Although this level of contrast is obviously problematic, less extreme variants using regions of the United States—for example, comparing areas with and without elevated water arsenic levels or farming and nonfarming regions—may be similarly if less extremely flawed. The use of spatial methods that discriminate within smaller units will generally be more effective in isolating the exposure of concern from other geographically based health influences because confounding may be less extensive and more amenable to analytic control. Modern computer-intensive techniques using GIS together with the availability of high-resolution health and demographic data for neighborhoods in the United Kingdom, United States, and other countries mean that an array of data to inform such small-area analyses are now available (Elliott and Wartenberg 2004; Jarup 2004; Nuckols et al. 2004).

In contrast with broad-scale geographic studies carried out at the country or regional level, spatial analyses at the small-area scale tend not to be purely ecologic, but to include a mix of individual-based and small-area data. This presents opportunities to mitigate ecologic bias and hence improve causal inference. For example, in a study of reproductive effects associated with landfill sites, individual data were available on the reproductive outcomes under investigation (birth weight, stillbirth, congenital anomalies), on the denominator (births), and on residential location (postcode of residence). For each birth, “exposure” was based on proximity to landfill sites, modeled within a GIS (residential location < 2 km from a landfill site). Meanwhile, information on potential confounding by socioeconomic variables and ethnicity was based on census data at small-area level (Elliott et al. 2001).

Interest has focused on “mixed” designs, that is, “aggregate” (Prentice and Sheppard 1995), “semi-individual” (Künzli and Tager 1997), and “semiecologic” (Richardson and Best 2003) designs, which combine individual- and group-level data to address problems of ecologic inference. As mentioned above, in applications from the U.K. Small Area Health Statistics Unit, typically individual data are available on health outcomes, and a mix of either individual or group data are available for environmental exposures and potential confounders (e.g., Elliott et al. 2001; Toledano et al. 2005; Wilkinson et al. 1999). Knowledge about the within-area distribution of individual exposure (and confounder) data allows proper model specification, to overcome the “pure specification bias” noted above and thus permit inference to be drawn about relationships at the individual level (Wakefield and Salway 2001). For example, information may be available from routinely available census (e.g., sample of anonymized records in the United Kingdom) or survey data (provided that appropriate geographic identifiers for the survey respondents can be obtained). Alternatively, purpose-designed surveys may be carried out on subsamples of the population in the areas under study. These could then not only provide information on the joint within-area distributions of the environmental exposures and confounders of interest, but also provide opportunity to carry out biologic sampling to help assess the validity of the exposure modeling. Random samples of sizes of the order of 10–50 up to 100–150 have been proposed, depending on the form of the exposure–response model, the expected causes of ecologic bias and the statistical model used for estimation (Glynn et al. 2008; Sheppard et al. 1996; Wakefield and Salway 2001). In our view, these mixed designs offer great promise for small-area analyses both to help validate the exposure modeling (reducing misclassification) and improve the model specification, and hence reduce bias.

Recently, variations on the mixed designs above have been proposed that combine group-level outcome and exposure/confounder data with data on covariates and health outcomes from small samples of individuals, “hybrid designs” (Haneuse and Wakefield 2007) and “hierarchical-related regression” (HRR) (Jackson et al. 2006). Such designs have the advantage of including linked exposure and health outcome data on the same individuals and can be thought of either in terms of an individual-level design supplemented by ecologic data to improve statistical power, or as an ecologic design supplemented by individual-level data to alleviate ecologic bias (Jackson et al. 2006). In practice, it is likely to be easier to obtain individual samples of covariate data for the mixed designs than to obtain suitable samples of linked exposure and health outcome data (especially for rare outcomes) for the hybrid/HRR designs. However, when such data do exist, the latter designs have the potential to greatly improve causal inference from geographic studies.

## Exemplar 1: Magnetic Fields from Overhead Power Lines and Childhood Cancer

The distribution and use of electricity are inherently spatial in that electricity is distributed by power lines from electricity-generating plants to substations and transformers, and ultimately to homes and workplaces. Each of those facilities has a location, and exposure is clearly determined in part by the position of the electricity-generating and distribution facilities relative to homes and other occupied locations. Electric utility companies are generally able to provide grid maps that show with reasonable precision where each element of the generation and distribution system is located. Furthermore, the health concerns with residential exposure to magnetic fields and cancer have focused on long-term exposure rather than moment-to-moment variation, allowing researchers to concentrate on where individuals live and work and not contend with the many briefly occupied microenvironments. Thus, exposure is spatially distributed, and human exposure is largely determined by stable structures such as homes and workplaces. All these elements seem promising for the application of spatial methods, although the utility of such methods has been limited to this point in time.

One clearly beneficial application of spatial methods has been in the selection of study areas for detailed investigation, targeting those with elevated prevalence of exposure. Whereas in the United States, distribution lines in urban areas have been the focus of concern and are the predominant determinants of household magnetic field exposure, in Europe, where distribution lines are buried, the effort has been to study residences that are proximal to high-voltage transmission lines. Such lines do not affect a large portion of the population, and thus studies that include broad regions are inefficient in having low exposure prevalence. Where studies have been conducted for other purposes—for example, the national study of childhood leukemia in the United Kingdom—the inefficiency in having an exposure prevalence of a few percent is tolerable (U.K. Childhood Cancer Study Investigators 1999), but studies designed specifically to address exposure to magnetic fields from high-voltage transmission lines can be designed with much greater efficiency. The idea is that very close to the transmission lines, magnetic fields from the power lines will predominate over other sources of extremely low-frequency electromagnetic radiation.

In a study of childhood cancer in Sweden, investigators began by isolating corridors around major transmission lines that were wide enough to encompass areas with and without elevated levels of magnetic fields (Feychting and Ahlbom 1993), and similar studies have been undertaken in other Scandinavian countries where high-resolution data on both residence and transmission lines are available (Olsen et al. 1993; Verkasalo et al. 1993). Assessment of exposure in the Swedish study ultimately required a home-by-home evaluation, but the prevalence of elevated exposure was optimized through the application of spatial methods. In the United Kingdom, a study focusing on proximity to overhead transmission lines (Draper et al. 2005) had the advantage that much larger potentially exposed populations were available than in the Scandinavian studies. A further study in the United Kingdom focusing on adult cancers is currently under way using the U.K. Small Area Health Statistics Unit database. Combining the initial application of spatial methods to define areas of potential exposure followed, where possible, with detailed evaluation of individual exposures may be generally useful where exposure prevalence varies regionally to a limited extent, and within region to a much greater extent.

In contrast to the European experience, in studies of neighborhood distribution lines in the United States, none has been able to effectively estimate individual exposures directly through the application of spatial methods, for multiple reasons. To do so would require a very fine level of resolution regarding power line configurations and residential locations of the order of a few meters, beyond the capability of most grid maps and home indicators, although recent advances in remote sensing, including satellite imagery, may achieve the required level of resolution (Bernstein and Di Gesù 1999). Beyond that, the specific occupied rooms of the home (e.g., location of child’s bedroom) would need to be considered. Selection of houses where cancer cases and controls have lived requires information on the occupants of the household (i.e., whether children are present), rarely available. Finally, the multiple research goals of most case–control studies require engaging the occupants directly, reducing the marginal cost of assessing magnetic fields directly and thus making this a more attractive approach than relying on spatial modeling or interpolation. Although it is theoretically possible to refine the power grid distribution maps to the point of automated assignment of exposure, including local topography, this seems unlikely to be effective given the many nuances of how exposure is determined and the lack of required information to classify exposure accurately. However, as has been done in Europe, spatial methods could provide a more refined strategy for selecting study locations in the United States based on pattern and prevalence of exposure, followed by conduct of detailed studies within those areas (Wartenberg et al. 1993).

## Exemplar 2: Drinking-Water Disinfection By-Products and Reproductive Effects

Research on health effects of drinking-water disinfection by-products offers perhaps greater promise for the more general application of spatial epidemiology methods than is the case with electromagnetic fields research. Concentration of drinking-water disinfection by-products is determined principally by the amount and character of organic material in the raw water combined with the treatment methods applied. This operates at the level of a water service area, which can range from dozens to hundreds of thousands of customers (up to 50,000 in the United Kingdom). Given the independent determination of drinking-water disinfection by-products from one supplier to the next, there can be large discontinuities in exposure levels across geographic areas, lending itself to study using the small-area approach (Keegan et al. 2001).

Although there are many ways in which exposure can vary within a water service area, including the use of tap water filters, water consumption, bathing and showering habits (King et al. 2004; Nuckols et al. 2005; Waller et al. 2001), and home ventilation, current evidence suggests that location alone remains a strong determinant of individual exposure (Keegan et al. 2001; King et al. 2004). In one study, a correlation of 0.73 was found between household trihalomethane concentration in the water and an estimate of total exposure to trihalomethanes based on ingestion, showering, and bathing habits (King et al. 2004).

**Figure 1:**
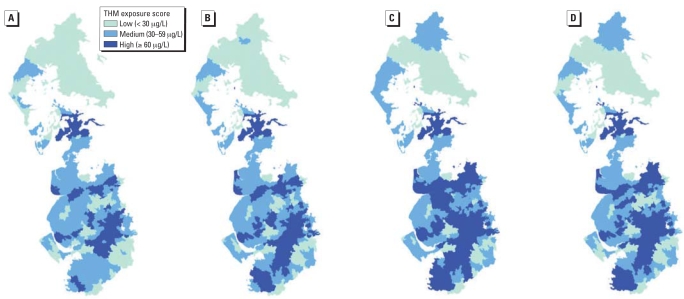
Maps showing water supply-zone-level total trihalomethane exposure categories by quarter: United Utilities Water, northwest England, 1997. (*A*) January–March. (*B*) April–June. (*C*) July–September. (*D*) October–December. Reproduced from Toledano et al. (2005) with permission from *Environmental Health Perspectives*.

The utility of the spatial approach to exposure estimation is illustrated in Figure 1. It shows the distribution of total trihalomethane concentrations (measured at household tap) across water zones (serving up to 50,000 people) in the United Utilities water area in northwest England over four 3-month periods (Toledano et al. 2005). The maps show large systematic spatial variation in trihalomethane concentrations across water zones, which is relatively consistent over the different seasons. Variation in trihalomethane concentrations was predominantly between rather than within zones, based on 15,984 observations in 288 water zones, from 1992 to 1996; for example, for chloroform, zone means (for 1996) ranged from 1.6 to 73.5 μg/L, and the median interquartile range within zones was 15.2 μg/L, compared with between-zone interquartile range of 26.8 μg/L (Keegan et al. 2001). Experience in the United Kingdom (Whitaker et al. 2005) and United States (Gallagher et al. 1998) has shown that incorporation of within- and between-community exposure variation can be integrated for application to the study of health outcomes.

There is, additionally, variation within the treatment distribution system due to varying residence times between the point of entry where the treated water is generated and the household taps. A number of sophisticated engineering models provide estimates of disinfection by-products (trihalomethanes and haloacetic acid) at various locations or nodes in the system (Sohn et al. 2004), potentially allowing the application of spatial methods on a somewhat more refined level. As is often the case, these more intensive and exacting efforts are not only more refined but also more subject to error and missing values (Gallagher et al. 1998). Given the need to provide adequate water pressure throughout the system and monitor the adequacy of such systems, the potential for spatial methods to capture exposure variability within treatment systems seems high and well worth continued exploration.

Finally, there are individual behavioral differences in water use, based on varying ingestion of hot and cold tap water (Nuckols et al. 2005), use of bottled water, and water use in locations other than the home. More refined still, the biologically effective dose may well be modified by genetic factors that affect metabolism and excretion of disinfection by-products. There is no obvious way in which spatial methods can be helpful even if there is some degree of predictability of drinking-water use based on location, for example, greater amounts in warmer climates. Although a number of studies have generated increasingly detailed information on the potential contribution of ingestion, bathing and showering, swimming, and other factors (Villanueva et al. 2007), the question for those designing and interpreting epidemiologic studies in the future is the extent to which water service area alone is an informative marker of exposure at a population level, and hence the degree of exposure misclassification to be expected if exposure assessment is based on area-level surrogate markers alone.

## Exemplar 3: Chronic Exposure to Air Pollutants and Cardiorespiratory Health

As noted above, exposure to air pollution is spatially determined and therefore should lend itself well to the geographic approach. Most of the epidemiology on air pollution and health has focused on the short-term effects of air pollution. In these studies, the variable of interest is time and space is “held constant”; that is, day-to-day fluctuations in health outcomes, such as hospital admissions or mortality, are correlated with fluctuations in air pollutant concentrations across whole areas or cities (Stieb et al. 2002). This is a very efficient and powerful design because potential confounding factors such as smoking and diet will not vary in aggregate from day to day.

Perhaps of more importance for public health, however, is whether chronic exposure to high levels of air pollutants is associated with adverse health effects. We can study this by investigating the association of air pollution with mortality or morbidity across different locations with varying levels of air pollutants. Although there is large variation across areas in the exposure variable (air pollutants) and limited within-area variability, both favorable features for spatial epidemiology, the study of chronic effects of air pollution faces a serious challenge due to confounding—although cities and neighborhoods may differ widely with respect to ambient air pollutant concentrations, they also tend to differ on myriad other variables that share the same spatial distribution as air pollution.

Several different designs using spatial methods have been used to investigate the chronic health effects of air pollution, while attempting to control the problem of potential confounding across areas. The Six Cities and American Cancer Society studies in the United States both used the semi-individual design (Künzli and Tager 1997) that compared city-level air pollution and mortality, but with data available on potential confounders at the individual level (Dockery et al. 1993; Krewski et al. 2000; Pope et al. 1995). This design gives excellent control for the individual-level confounders (including smoking) measured in the cohorts, although the analyses conducted across cities could still be susceptible to cross-level confounding and bias (Webster 2002).

A cohort study in the Netherlands used spatial methods to exploit the large differences in potential exposure to air pollutants according to distance from major roads. An indicator variable was used to classify individuals according to chronic exposure to traffic-related air pollutants based on place of residence. The study was then analyzed as an individual-based cohort in the usual way (Beelen et al. 2008; Hoek et al. 2002).

**Figure 2:**
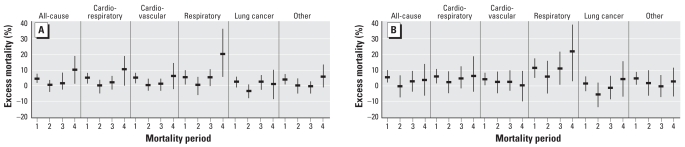
Excess relative risks (≥ 30 years of age) for different mortality periods with exposure data for the preceding 0–4 years for black smoke (per 10 mg/m^3^) (*A*) and sulfur dioxide (per 10 ppb) (*B*), for wards in Great Britain with a fixed-site air pollution monitor. Mortality periods: 1, 1982–1986; 2, 1986–1990; 3, 1990–1994; 4, 1994–1998. Reproduced from Elliott et al. (2007) with permission from BMJ Publishing Group.

In the United Kingdom, census wards (5,000 residents on average) where a fixed-site monitor was located were selected to analyze geographic associations between air pollutants (black smoke and sulfur dioxide) and mortality at small-area scale. This design minimized within-area variability in the exposure variables and maximized between-area variability, a key feature for the successful application of small-area methods, as already noted. However, a limitation compared with the U.S. and Dutch studies was that information on individual-level confounders was not available; instead, control for confounding across areas was carried out by use of a deprivation score (closely correlated with smoking rates) and a measure of rurality at the small-area scale (Elliott et al. 2007). There appeared to be adequate control for confounding by smoking because the association of lung cancer mortality with air pollution disappeared after adjustment. The study showed, per 10 ppb sulfur dioxide, 4.2%, 5.2%, and 3.5% excess risk of all-cause, cardiorespiratory, and all other cause mortality, respectively, compared with 5%, 6%, and 5% in the American Cancer Society study (Krewski et al. 2000). The largest excess relative risks in the U.K. study among all causes of death investigated were found for respiratory mortality, especially for levels of air pollutants measured in the previous 4 years, and in the most recent period of mortality studied (1994–1998) (Figure 2). Overall, adjusted excess relative risk for respiratory mortality across all periods combined was 3.6% [95% confidence interval (CI), 2.6–4.5%] per 10 μg/m^3^ black smoke and 13.2% (95% CI, 11.5–14.9%) per 10 ppb sulfur dioxide, and in the most recent period (1994–1998) it was 19.3% (95% CI, 5.1–35.7%) and 21.7% (95% CI, 2.9–38.5%), respectively. In the latest report from the Dutch study, highest excess risk was also found for respiratory disease mortality, 22% (95% CI, –1% to 50%) per 10 μg/m^3^ black smoke.

These studies provide good examples of how spatial epidemiologic methods can be used to exploit large between-area differences in environmental exposures (air pollution), with careful attention to issues of confounding and potential biases. Despite the design differences, ranging from individual-level (Dutch study) through semi-individual (U.S. studies) to ecologic study across small areas (United Kingdom), results were consistent in finding associations of chronic exposure to air pollutants with mortality. As indicated above, effect sizes between studies also showed some consistency (though for some analyses there were also differences) strengthening plausibility. Together, their findings add to the evidence that air pollution has long-term effects on mortality and point to continuing public health risks, even at the relatively lower levels of pollution that now occur.

## Discussion

Among epidemiologists, there is in our view well-justified enthusiasm regarding the potential benefits from the application of spatial methods to identify environmental contributors to the etiology of disease. However, the effectiveness of these methods depends not just on the enthusiasm or creativity of the investigator or on the sophistication of the technology, but also on the manner and extent to which the environmental agents happen to be “cooperative” in their spatial distribution and in offering clear, useful indicators of that distribution.

In some instances, the exposure simply does not vary spatially in a way that makes location alone a useful proxy of exposure. Some exposures are largely determined by individual behavior, such as the use of household pesticides. Some exposures vary spatially but at the level of an individual home or rooms within a home, for example, the presence of molds. Under such conditions, at best, neighborhoods or regions that have varying prevalence of such exposures can be identified as a predictor of individual exposure. However, the goal is not to simply predict or assess whether location is associated with exposure but to use location as a surrogate marker of exposure, a much higher standard calling for a much stronger relationship to be informative.

Some exposures that are determined spatially can be so extensively altered among individuals within the area as to limit or even negate the effectiveness of the geographic indicator. For example, exposure to indoor radon is ultimately determined by local geology and should therefore be amenable to spatial analysis methods, but exposure is so strongly affected by home construction and ventilation (Gunby et al. 1993) as to dilute or eliminate the potential for using region as a marker of individual exposure. Exposure sources may vary geographically, for example, hazardous waste sites or cellular telephone communication towers, but actual exposure potential may vary greatly from site to site. Thus, mere proximity to such sources may be a limited or poor proxy for exposure of individuals near any particular site—although, in aggregate, these studies may give indication where further, more detailed investigations might be warranted (Elliott et al. 2001).

Both chlorination by-products and outdoor air pollution are examples where strong spatial contrasts between areas make these suitable for study by the spatial epidemiologic approach. For other exposure scenarios, a case-by-case judgment will need to be made as to the extent that individual behaviors or microenvironments may predominate over broader-scale spatial variations in exposure, to determine the utility of undertaking an investigation using the small-area approach. This judgment may need to be informed by initial small-scale pilot studies to investigate pathways, magnitude, and extent of individual exposures, potentially to include biologic as well as environmental or personal sampling.

Our exemplars were chosen to illustrate how spatial epidemiologic methods have been used in practice to address some important issues in environmental epidemiology. In the electromagnetic fields example, spatial methods have been used to increase efficiency and study power by identifying areas with high prevalence of exposure. In the study of chlorination by-products, the large between- to within-area variability was exploited to maximize exposure contrasts, as was the case in the air pollution example (where potential for between-area confounding was also a particular concern).

From this review of design issues in small-area studies, several common concepts and themes emerge:

- Exposures to environmental agents (as well as social and behavioral influences on disease) are often similar within small geographic units and diverge more dramatically across larger regions. The ability of epidemiologic studies to determine whether the putative causal agent is capable of causing disease rests to a great extent on the magnitude of exposure contrast that can be included across diverse geographic areas within a given study.
- Accurate assignment of exposure depends on the availability of effective markers of variation in exposure, whatever the underlying diversity of exposures may actually be. The ideal situation involves subsets of the population with clearly defined discriminating markers, for example, “smoker” versus “non-smoker”; and in some instances but not others, location can serve as such a marker.
- The question is not whether location is a predictor of exposure (often it clearly is), but rather, what is the quantitative impact of location in conjunction with other determinants of exposure? That is, to what extent is geographic location alone an effective marker of individual exposure? In order to address this issue, research needs to examine the full spectrum of individual and group influences on exposure in order to quantify the range of contributors in large populations.
- There is often a trade-off between population size and the quality and detail of information on exposure that is available. Very detailed study of small areas that have experienced major pollution episodes, for example, Woburn, Massachusetts (Costas et al. 2002), and Toms River, New Jersey (Maslia et al. 2005), seek to determine whether there is a cause-and-effect relationship between exposure and disease among the residents of the area. Intensive attempts are made to draw inferences based on small case groups through the application of extremely sophisticated and informative spatial methods at the level of neighborhoods or households. No matter how elegantly such studies are done, however, there is a severe, inherent limitation in the inferences that can be drawn based solely on the size of the population available.
- Large populations are needed to study many of the health concerns of greatest interest in environmental epidemiology, such as cancer or birth defects. To generate adequately precise estimates of the magnitude of association between environmental agents and rare events, very large populations must be assembled, often requiring aggregation of multiple subunits. An example is the study of cancer incidence near radio and television transmitters in the United Kingdom, where identification of a potential “cluster” of leukemias near one transmitter was followed up by investigating cancer incidence near all such transmitters in Great Britain (Dolk et al. 1997a, 1997b). Such a pool of many similarly polluted sites may not exist, the idiosyncrasies of the exposure circumstances may preclude such pooling, or the effort required for detailed spatial analysis of multiple sites may be prohibitive.
- Routine systems that can rapidly collate health and other data from many locations, as is available in the United Kingdom through the Small Area Health Statistics Unit, can greatly facilitate the process of pooling across sites (Aylin et al. 1999; Jarup 2004) and identify questions or areas for more detailed study. Such detailed follow-up studies are inevitably expensive and time-consuming, so great efficiencies can be gained through initial investigation by use of the small-area approach.
- For chronic diseases including most cancers, latency effects are important, such that exposures experienced many years previously, or accumulated exposures, may be crucial. Under these circumstances, present day measurement or monitoring, or present-day location, may be a poor marker of the exposure metric of most interest. Thus, long-term residential (and often occupational histories) may be important, and these may not be captured well in routine systems.
- Semi-individual/semiecologic designs that combine data on the general population with individual-level survey data offer an attractive way to improve model specification and hence inference in small-area studies. Clearly, we cannot apply these methods to large numbers of people, but collection of such data even on small subsamples of the population will provide valuable additional information, for example, on within-area variability of both the exposure data and potential confounders, which will help improve the exposure estimation and modeling (Jackson et al. 2006). This in turn should lead to improved assessment of risk.

In conclusion, spatial methods not only must be able to assign exposure accurately, but also must be able to do so for geographic areas with differing exposure potentials. These methods will be most useful when no other compelling reasons exist for collecting individual-level data through interview or collection of biologic specimens, or where such studies are considered infeasible or prohibitively expensive. In these circumstances, spatial epidemiologic studies based on readily available (ecologic and individual) sources of data can give clues or hints as to which areas or pollutants might be worthy of further study using more detailed individual designs. When in-person data collection is required, there are often efficient approaches to collecting data that are superior to geographically based exposure estimates through self-report, environmental measurements, or biologic monitoring, although this might not always be the case, for example, where historical exposure estimates are required. Data from small subsamples of the population, based on either existing or purpose-designed surveys, can also be used to enhance the design of spatial studies and improve inferences that can be drawn from them. The effectiveness of these methods calls for research that includes both spatial and nonspatial determinants of exposure in order to evaluate empirically and in quantitative terms the adequacy of location, or modeled estimates, as a proxy for individual exposure. Ultimately, the aim is to contribute new knowledge and insights into the etiology of diseases and ill-health related to exposure to environmental pollutants.
